# Efficacy and safety of early supplementation with 800 IU of vitamin D in very preterm infants followed by underlying levels of vitamin D at birth

**DOI:** 10.1186/s13052-017-0361-0

**Published:** 2017-05-04

**Authors:** Sang Yeun Cho, Hyun-Kyung Park, Hyun Ju Lee

**Affiliations:** Department of Pediatrics, Hanyang University Seoul Hospital, 17 Haengdang-dong, Seongdong-gu, Seoul 133-792 South Korea

**Keywords:** Vitamin D, Supplementation, Premature infants, Follow-up, DXA

## Abstract

**Background:**

To determine the efficacy and safety of early supplementation with 800 IU of vitamin D in very low birth weight (VLBW) infants.

**Methods:**

Sixty-six infants with a birth weight less than 1500 g admitted to the Neonatal Intensive Care Unit. Of these, 52 infants were eligible and received 800 IU/day vitamin D from 2 weeks of age. We examined 25-hydroxyvitamin-D (25[OH]D) levels from cord blood at birth and serum at 32 and 36 weeks of postmenstrual age.

**Results:**

The study infants were divided by cord-blood levels of 25(OH)D at birth into 25(OH)D concentrations < 10 ng/mL (*n* = 20) or ≥ 10 ng/mL (*n* = 29). Vitamin D intake of 800 IU/day safely achieved an 88% probability of vitamin D sufficiency at 36 weeks postmenstrual age in VLBW infants with cord-blood levels of 25(OH)D ≥ 10 ng/mL, and 65% probability of vitamin D sufficiency was observed in infants with 25 OHD concentrations < 10 ng/mL at birth.

**Conclusion:**

Considering the efficacy and safety of vitamin D supplementation in this study, vitamin D intake of 800 IU/day may enhance vitamin D status during early hospitalization in VLBW infants with 25 OHD concentrations < 10 ng/mL at birth. The clinical significance of optimal vitamin D intake in VLBW infants needs to be studied in larger controlled studies.

## Background

Vitamin D deficiency is common among preterm infants due to difficulties in adequate enteral nutrition and lack of sunlight exposure during hospitalization. Furthermore, very low birth weight (VLBW) infants often have less time to store vitamin D from mother due to a decreased trans-placental transfer and may also have an increased vitamin D requirement. Due to the potential impact of vitamin D on many developmental processes in preterm infants, Jobe et al. [1] emphasized the fundamental benefits of vitamin D treatment for multiple developmental abnormalities, referring to the high percentage of preterm infants with blood vitamin D levels < 20 ng/mL [2]. However, no consensus recommendation for vitamin D deficiency was made specifically for preterm infants, because few data on bone mineralization rates *in utero* are reported. The European guideline on daily vitamin D supplementation recommend 800 to 1000 IU/day for preterm infants, while western countries recommend 400 IU/day [3].

Although the incidence of vitamin D insufficiency in preterm infants varies according to different geographical locations, mean serum 25(OH)D levels during 1–3 days after birth in preterm infants ranges from 9.5 to 25 ng/mL in both Europe and Western world [4–7]. Considering the high incidence of vitamin D deficiency/insufficiency in preterm infants that miss the late gestation period, which builds vitamin D stores from mother to fetus, a vitamin D supply might be necessary and urgent to compensate the sufficient vitamin D level. While breast milk feeding is currently recommended as a source of early nutrition in full and preterm infants, an infant diet with breast milk alone does not contain enough vitamin D. The definition of vitamin D sufficiency in preterm infants is still controversial; optimal vitamin D status is based on the vitamin D concentrations for multiple health outcomes in adults with a target value for 25(OH)D levels >30 ng/ml [8]. Responses to different doses of vitamin D supplementation and monitored supplementation of vitamin D with serial measurement of 25 (OH)D concentrations are limited in VLBW within a month after birth. Nevertheless, the appropriate amount of vitamin D required for optimal growth in VLBW is still important issue to address. The aim of this study was to determine whether daily vitamin D supplementation with a high dose of 800 IU is safe and effective to increase the proportion of VLBW infants with 25(OH)D > 30 ng/mL, from 2 weeks of age until hospital discharge.

## Methods

### Study population

Preterm infants with a birth weight less than 1500 g admitted to the Neonatal Intensive Care Unit of Hanyang University College of Medicine and born between March 2014 and October 2015 were enrolled in this study. We excluded subjects with major congenital and choromosomal abnormalities. Infants who were transferred, intolerant of feeds, died before first assessment or for whom consent was not obtained were also excluded. This study included written informed consent and was approved by the Hanyang University Institutional Review Board.

### Study protocol

At birth, serum calcium, phosphorus, alkaline phosphatase and 25(OH)D concentrations were obtained from cord blood samples before vitamin D supplementation was started. Early vitamin D supplements (Sunny D, GMP Laboratories of America, Inc., Anaheim, CA, USA) were initiated at 14 days after birth at a dose of 800 IU/day through an orogastric tube just before feeds, if infants were tolerating trophic feeds. Trophic feedings were initiated 1–3 days after birth and increased by 10–20 mL/kg/d. Feeding volume and advancement, and feeding intolerance were determined and monitored by neonatologists based on the same protocol. After receiving 100 m L/kg per day of enteral feedings, fortified human milk (including 82 mg calcium, 45 mg phosphorus and 100 IU vitamin D per 100 ml) or a mineral-fortified preterm formula (including 115 mg calcium, 62 mg phosphorus and 66 IU vitamin D per 100 ml) were used for nutritional care. The total content of daily vitamin D intake was estimated and cumulative dose in human milk fortifier or formula did not exceed 900 IU/day in the present study. Vitamin D supplementation continued from two weeks until 36 weeks postmenstrual age (PMA), and was discontinued if surgical necrotizing enterocolitis or bowel perforation was diagnosed or 25(OH)D concentrations > 80 ng/mL were detected. We measured 25(OH)D levels from cord blood at birth and serum at 32 and 36 weeks PMA. Eligible infants were stratified by cord-blood levels of 25(OH)D into two groups, < 10 ng/mL and ≥ 10 ng/mL. We determined the efficacy and safety of early supplementation with 800 IU vitamin D.

### Outcome measures

The primary outcome measured the proportion of vitamin D sufficiency (>30 ng/mL) at 32 and 36 weeks PMA and was analyzed by cord blood 25(OH)D levels (<10 ng/mL versus ≥ 10 ng/mL) at birth. Vitamin D deficiency was categorized as 25(OH)D levels < 10 ng/mL. The 25(OH)D cutoff of 30 ng/mL was chosen based on the earlier study, in which this level was considered to be vitamin D sufficiency [9]. Secondary outcomes included vitamin D toxicity profile: serum levels of calcium, phosphorus, alkaline phosphatase, the proportion of vitamin D excess and parathyroid hormone (PTH) at 32 weeks PMA; hypercalciuria (urine calcium to creatinine ratio [UCa/UCr] > 0.8 mg/mg) or nephrocalcinosis at 36 weeks PMA. Bone mineralization was assessed at 36 weeks PMA by total body densitometry using a commercially available DEXA (Hologic QDR-4500; Hologic, Waltham, Mass., USA) in infant whole-body mode (QDR software for Windows XP, version 12.7; Hologic), with an exam performed at hospital discharge. DEXA evaluated bone mass of the total body and of the lumbar spine (S) (L2-L4), lean mass (LM, g), and fat mass (g). Bone mineral density (mg/cm^2^) was obtained by dividing bone mineral content (g) by the projected bone area (BA, cm^2^). Data on maternal age, gestational diabetes mellitus, antenatal steroid use, histologic chorioamnionitis, maternal education, birth during vitamin D synthetizing period, gestational age, birth weight, small gestational age, sex, type of delivery, and Apgar scores at 1 and 5 min were retrieved for each infant. To evaluate the effect of the course of prematurity on vitamin D status, we evaluated respiratory distress syndrome (RDS), patent ductus arteriosus (PDA), necrotizing enterocolitis (NEC ≥ grade 2), sepsis (clinical or proven), bronchopulmonary dysplasia (BPD, ≥ moderate), retinopathy of prematurity, intraventricular hemorrhage (IVH ≥ grade 2), type of infant formula, ventilation days, hospital days and total parenteral nutrition days. The diagnosis and severity of BPD was based on the need for supplementary oxygen at 28 days of age and 36 weeks PMA [10]. Intraventricular hemorrhage was classified according to Volpe [11]. Necrotizing enterocolitis was defined according to Bell staging criteria [12]. Chorioamnionitis was defined by the presence of histologic chorioamnionitis or umbilical cord vasculitis of grade 2 or greater, using the grading system suggested by Salafia et al. [13].

### Statistical analysis

The sample size for this study was estimated based on an earlier study, which observed the positive outcome on vitamin D levels in high-dose groups (960 IU/day), with a power of 80% (α = 0.05, δ = 5 IU/L, pooled SD = 12 IU/L, *N* = 25 infants per group) [5]. Statistics were calculated with Student’s t-tests and Chi-square tests for means and frequencies, respectively. Numeric data are presented as mean and standard deviation, as the data were normally distributed. Calculations were performed with SPSS software version 17.0 (SPSS, Chicago, IL, USA). *P* values less than 0.05 were considered statistically significant.

## Results

**Fig. 1:**
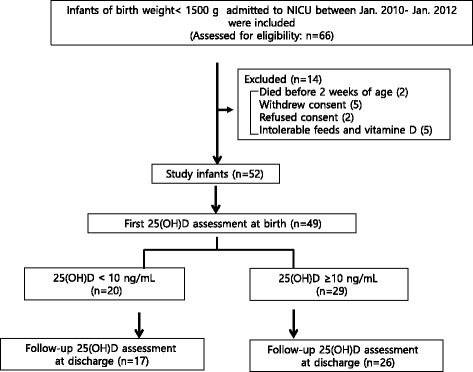
Study flow

**Fig. 2:**
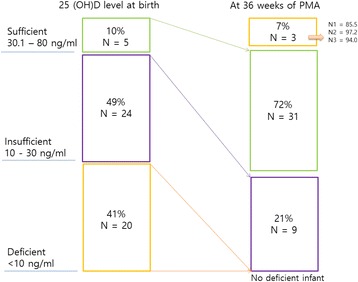
Changes in vitamin 25(OH)D levels from birth to 36 weeks postmenstrual age

We enrolled the 66 infants with a birth weight less than 1500 g who were born during the study period. Of these, 2 infants died before 2 weeks of age; consent was refused by 2 parents and withdrawn after decision by 5 parents shortly; 5 infants were intolerant of trophic feeds and vitamin D. Thus, a total of 52 infants were eligible and completed the intervention after parents provided informed consent. We check 25(OH)D levels at birth in 49 infants and at 36 weeks PMA in 43 infants (Fig. 1). Among the study groups at baseline, the number of cases of vitamin D deficiency and insufficiency were 20 (41%) and 24 (49%), respectively, while after vitamin D supplementation, the number of cases of vitamin D deficiency and insufficiency were 0 (0%) and 9 (21%) at 36 ± 1 weeks PMA. Five infants (10%) had sufficient 25(OH)D levels in range of 30.1–80 ng/ml at birth, and 31 infants (72%) at 36 ± 1 weeks PMA achieved sufficient 25(OH)D levels after vitamin D supplementation. Three preterm neonates had 25(OH)D levels over 80 ng/ml (85.5, 94 and 97.2 ng/mL; Fig. 2).

The study population was comprised of 52 premature newborns (26 boys and 26 girls) with a mean gestational age of 27.1 ± 2.5 weeks. Eight (15.4%) newborns had a birth weight under the 10th percentile. Regarding neonatal prematurity-related diseases, 25 (48%) patients presented with bronchopulmonary dysplasia (≥ moderate), 3 (6%) with necrotizing enterocolitis, and 6 (12%) with IVH (≥ grade2). We classified patients into two groups based on cord blood 25(OH)D levels. A total of 52 infants were eligible but the study groups was divided and analyzed by cord blood 25(OH)D levels from cord blood in 49 infants, excluding 3 infants who is missing cord 25(OH)D assessment at birth. Demographic and clinical characteristics of the study population were comparable between cord 25(OH)D concentrations < 10 ng/mL and cord 25(OH)D concentrations ≥ 10 ng/mL. No significant differences existed in demographics between the two groups. The prevalence of cord 25(OH)D concentrations < 10 ng/mL was significantly higher in the presence of a GDM mother than in a non-GDM mother (*P* = 0.035). However, neonates born during the Vitamin D synthesis period, from April to October (20 neonates, 69%), had higher 25(OH)D concentrations in cord blood samples than those not born during the vitamin D synthesis period (7 neonates, 35%; *P* = 0.023; Table 1).

**Table 1:** Demographic characteristics

Characteristics	All patients (*n* = 52)	Cord 25(OH)D concentrations <10 ng/mL (*n* = 20)	Cord 25(OH)D concentrations ≥10 ng/mL (*n* = 29)
Maternal age, years	34.3 ± 3.4	32.4 ± 3.1	33.8 ± 4.0
GDM	6 (12%)	5 (25%)	1 (3%)^*^
Preeclampsia	4 (8%)	0 (0%)	3 (10%)
Antenatal steroid	44 (85%)	19 (95%)	22 (79%)
Histological Chorioamnionitis	33 (64%)	11 (55%)	21 (72%)
Completed college	38 (73)	13 (65%)	24 (83%)
Birth during vitamin D synthesizing period	30 (58%)	7 (35%)	20 (69%)^†^
Gestational age (weeks)	27.1 ± 2.5	27.5 ± 2.5	26.8 ± 2.6
Small gestational age	8 (15%)	2 (10%)	5 (17%)
Birth weight (g)	971.2 ± 252.4	1045.5 ± 271.8	933.5 ± 203.6
Birth length (cm)	34.1 ± 3.5	34.3 ± 3.4	34.1 ± 3.6
Birth head circumference (cm)	24.6 ± 2.2	24.8 ± 2.3	24.5 ± 2.2
Male sex	26 (50%)	7 (35%)	16 (55%)
Cesarean section	40 (77%)	18 (90%)	20 (71%)
Apgar score at 5 min	5.1 ± 1.5	5.3 ± 1.7	4.9 ± 1.3
RDS	47 (90%)	17 (85%)	27 (96%)
PDA	35 (67%)	15 (79%)	19 (66%)
NEC	3 (6%)	0 (0%)	2 (7%)
Culture-proven sepsis	17 (33%)	5 (26%)	11 (38%)
BPD (≥ moderate)	25 (48%)	7 (37%)	16 (55%)
ROP (≥ stage 2)	24 (46%)	8 (40%)	14 (50%)
IVH (≥ grade 2)	6 (12%)	4 (20%)	2 (7%)
Breast milk with/without formula	38 (73%)	13 (65%)	22 (76%)
Exclusively fortified breast milk	13 (25%)	5 (25%)	8 (28%)
Ventilation days (d)	24.1 ± 18.4	20.9 ± 18.1	20.9 ± 18.1
Hospital days (d)	85.5 ± 33.2	77.6 ± 24.8	86.6 ± 28.3
TPN days (d)	47.1 ± 32.5	37.2 ± 22.7	47.2 ± 22.4

Data are presented as n (%), median (range), or mean ± SD

*GDM* gestational diabetes mellitus, *RDS* respiratory distress syndrome, *PDA* patent ductus arteriosus, *NEC* necrotizing enterocolitis, *BPD* bronchopulmonary dysplasia, *ROP* retinopathy of prematurity, *IVH* intraventricular hemorrhage, *TPN* total parenteral nutrition

^*^ *P* = 0.035 for the difference between cord 25(OH)D concentrations < 10 ng/mL vs. ≥ 10 ng/mL

^†^ *P* = 0.023 for the difference between cord 25(OH)D concentrations < 10 ng/mL vs. ≥ 10 ng/mL

All neonates received 800 IU 25(OH)D per day from first 2 weeks of age. We checked serum vitamin D at birth, 32 ± 1 weeks PMA and 36 ± 1 weeks PMA. Vitamin D status is presented in Table 2. After the vitamin D supplementation, the serum 25(OH)D status at discharge in the two groups converged into increasing 25(OH)D concentrations. Nevertheless, serum 25(OH)D levels at 36 ± 1 weeks PMA were significantly lower in the infants with cord 25(OH)D concentrations < 10 ng/mL compared with those with cord 25(OH)D concentrations ≥ 10 ng/mL (43.1 ± 20.3 ng/mL versus 57.7 ± 21.9 ng/mL; *P* = 0.03). The proportion of infants with vitamin D sufficiency was lower in those with cord 25(OH)D concentrations < 10 ng/mL than in those with cord 25(OH)D concentrations ≥ 10 ng/mL at 36 ± 1 weeks PMA (65% vs. 88%; *P* = 0.12; Table 2).

**Table 2:** Vitamin D status

Variable	Cord 25(OH)D concentrations <10 ng/mL	Cord 25(OH)D concentrations ≥10 ng/mL	*P*
At birth			
N	20	29	
Serum 25(OH)D levels	8.3 ± 1.9	21.4 ± 8.5	<0.001
Vitamin D sufficiency (>30 ng/mL)	0 (0%)	5 (17%)	0.07
32 ± 1 weeks postmenstrual age			
N	13	18	
Serum 25(OH)D levels	15.3 ± 10.1	24.1 ± 9.3	0.05
Vitamin D sufficiency (>30 ng/mL)	3 (23%)	4 (22%)	1.00
36 ± 1 weeks postmenstrual age			
N	17	26	
Serum 25(OH)D levels	43.1 ± 20.3	57.7 ± 21.9	0.03
Vitamin D sufficiency (>30 ng/mL)	11 (65%)	23 (88%)	0.12

Data are presented as n (%), median (range), or mean ± SD

Table 3 presents the levels of serum calcium, phosphorous, alkaline phosphatase level at birth, 32 ± 1 weeks PMA, and 36 ± 1 weeks PMA as markers of vitamin D status. The PTH levels at 32 ± 1 weeks PMA were not significantly different between the groups (76.3 ± 50.1 versus 74.2 ± 69.1 pg/mL; *P* = 0.92). We assessed urine calcium and creatinine, and used abdominal ultrasound to determine nephrocalcinosis and bone mineral density at 36 ± 1 weeks PMA. Three of 49 (6%) neonates who received 800 IU 25(OH)D had nephrocalcinosis. The two infants of the three infants with nephrocalcinosis were not related with both excessive amounts of vitamin D and an increased UCa/UCr at 36 ± 1 weeks PMA. One of the three infants with exceeding level of vitamin D had both nephrocalcinosis on a renal ultrasound and increased UCa/UCr > 0.8. The three infants in the cord 25(OH)D concentrations ≥ 10 ng/mL group had a vitamin D excess (85.5, 94 and 97.2 ng/mL) at 36 ± 1 weeks PMA.

**Table 3:** Secondary outcome variables at 32 and 36 weeks postmenstrual age

Variable	Cord 25(OH)D concentrations <10 ng/mL (*N* = 20)	Cord 25(OH)D concentrations ≥10 ng/mL (*N* = 29)	*P*
At birth			
Serum calcium, mg/dL	7.0 ± 0.7	7.3 ± 0.8	0.12
Serum phosphorus, mg/dL	5.0 ± 1.4	4.8 ± 1.4	0.51
Serum ALP, IU/L	205.6 ± 63.7	178.8 ± 49.1	0.10
32 ± 1 weeks PMA			
Serum calcium, mg/dL	9.7 ± 0.5	9.4 ± 0.7	0.86
Serum phosphorus, mg/dL	5.9 ± 0.9	5.2 ± 0.8	0.13
Serum ALP, IU/L	366.5 ± 102.3	422.0 ± 138.5	0.05
Serum PTH, pg/mL ^*^	76.3 ± 50.1	74.2 ± 69.1	0.92
Vitamin D excess (>80 ng/mL)	0	0	-
36 ± 1 weeks PMA			
Serum calcium, mg/dL	9.7 ± 0.4	9.7 ± 0.6	0.51
Serum phosphorus, mg/dL	6.2 ± 0.6	5.7 ± 1.9	0.07
Serum ALP, IU/L	284.6 ± 91.6	338.8 ± 127.1	0.11
Vitamin D excess (>80 ng/mL)	0/17	3/26	0.27
UCa/Cr	0.13 ± 0.16	0.19 ± 0.28	0.50
UCa/Cr >0.8	0	1 (3%)	1.00
Nephrocalcinosis, n	1 (5%)	2 (7%)	1.00
Weight, g	1881.1 ± 254.1	1813.2 ± 331.3	0.44
Length, cm	41.3 ± 1.5	40.5 ± 2.2	0.19
Head circumference, cm	30.6 ± 1.2	29.6 ± 1.4	0.43
BMC, g^†^	2.6 ± 2.0	2.1 ± 1.7	0.44
BMD, g/cm^†^	0.11 ± 0.02	0.10 ± 0.02	0.63

*Data are presented as n (%), median (range), or mean* ± SD unless otherwise indicated. -, could not be estimated because the data is skewed or categorical, or there are no events in both the groups

*ALP* alkaline phosphatase, *PMA* postmenstrual age, *PTH* parathyroid hormone, *BMC* bone mineral content, *BMD* bone mineral density

^*^ *n* = 18 and 20 in the cord 25(OH)D concentration < 10 ng/mL and ≥ 10 ng/mL groups, respectively

^†^ *n* = 12 and 19 in the cord 25(OH)D concentration < 10 ng/mL and ≥ 10 ng/mL groups, respectively

With the exception of these three patients, there were no observed cases of hypervitaminosis, hypercalcemia, hypercalciuria, polyuria, dehydration or nephrocalcinosis. DEXA was performed in 63% (31 of 49) of the infants at 36 ± 1 weeks PMA. BMD was comparable (0.11 ± 0.02 versus 0.10 ± 0.02; *P* =0.63). There were no differences between the groups regarding growth (Table 3).

## Discussion

This prospective study was planned to determine whether 800 IU vitamin D supplementation from 2 weeks of age is safe and effective to reach sufficient vitamin D levels at 36 weeks PMA in VLBW infants. Among VLBW infants with 25(OH)D concentrations < 10 ng/mL at birth, none had an excess level of 25(OH)D >80 ng/ml after 800 IU vitamin D supplementation, although 35% of infants with vitamin D deficiency from birth were still vitamin D insufficient at 36 weeks PMA. The response to vitamin D supplementation was dependent on the baseline vitamin D stores at birth. While the use of 800 IU/d of vitamin D achieved serum 25(OH)D concentrations to the adequate range of 30–80 ng/mL without toxicity at 36 weeks PMA, this dose may still be too low to restore vitamin D stores for the majority of infants with 25(OH)D concentrations < 10 ng/mL at birth.

Vitamin D is important for supporting a large number of physiological processes such as bone mineralization, immune modulatory functions and early lung development. Recent studies demonstrated that low vitamin D predispose to increased risk of rickets, immune dysfunction, asthma, type 1 diabetes and viral respiratory tract infections during infancy [14–17]. The potential benefits derived from adequate vitamin D levels warrant assessment the vitamin D levels in preterm infants. Moreover, inadequate vitamin D levels in cord blood are associated with immune status and the prevalence of severe respiratory tract infections in the first year of life, as well as reduced bone density during childhood [18–20]. Serial measurements of serum 25(OH)D to evaluate vitamin D status during hospitalization may help to identify preterm infants at risk of vitamin D deficiency. Considering the high prevalence of vitamin D deficiency in pregnant mothers, sufficient vitamin D supply in preterm infants could be important to quickly correct the fetal low plasma levels of vitamin D. A vitamin D intake of 800 IU to 1000 IU per day during first months of life is recommended based on European guidelines [21]. It is accepted that an intake of 800 IU to 1000 IU per day would improve serum 25(OH)D status and, subsequently, the calcium absorption rate, preventing osteopenia among preterm infants [22]. However, the exact and efficient timing of vitamin D-dependent absorption of calcium remains unknown in preterm infants. Preterm infants with radiologic evidence of rickets or high alkaline phosphatase (>800 IU/L) are generally given the upper intake total of 800 IU/day of vitamin D. Despite vitamin D supplementation, it is difficult to maintain the recommended daily vitamin D intake from dietary in preterm infants, and there are few evidence-based data to support vitamin D supplementation to meet a desired serum 25(OH)D according to vitamin D status at birth in preterm infants. This lack of evidence has resulted in varying practices among neonatologists, and controversy remains about the thresholds and strategies for vitamin D intake of 800 IU among VLBW infants earlier in life.

Consistent with previous reports about a dose-response effect of vitamin D supplementation [2, 23], we observed that early supplementation with 800 IU vitamin D safely achieved an 88% probability of vitamin D sufficiency at 36 PMA in the majority of infants with cord-blood levels of 25(OH)D ≥ 10 ng/mL. Although a number of vitamin D deficiency was significantly lower after supplementation than before supplementation in the present study, supplementation with 800 IU might not be sufficient to achieve optimal vitamin D levels in preterm infants with underlying level of vitamin D deficiency from birth. The different response to vitamin D supplementation could be explained by VDR gene polymorphism involved in vitamin D receptor, which results in vitamin D deficiency at 36 PMA in some babies after supplementation [24]. Recently, studies have observed that circulating 25(OH)D levels are related with the potential genes such as VDR, CYP2R1 and VDBP [24–26]. However, recommended oral vitamin D supplementation to attain adequate serum 25(OH)D concentrations has not been recognized in VLBW infants with underlying level of vitamin D deficiency from birth. Recently, Natarajan et al. [23] reported that daily supplementation of vitamin D in doses of 800 IU compared with 400 IU appears to reduce the prevalence of vitamin D deficiency at 40 weeks PMA in preterm infants of 28 to 34 weeks gestation. A randomized controlled trial in 100 infants with gestational age between 23 and 27 weeks demonstrated that the 800 IU/d dose of vitamin D supplementation prevented vitamin D deficiency, without toxicity, with concentrations above the desired 25(OH)D range of 20–60 ng/ml in the majority of infants on day 28 [2]. Grant et al. [9] established the 800 IU/d dosage of vitamin D met the 25(OH)D > 30 ng/mL in 81% of study infants at the age of 3 months, while the 400 IU/d dosage of vitamin D sustained those concentration in 55% of study infants. Cameron C. et al. [15], in a randomized controlled prospective trial of full-term infants, suggested that serum 25(OH)D concentrations were greater in the higher-dose group of 800 IU/d than in the lower-dose group of 400 IU/d supplementation until 6 months of age. Daily maternal (2000 IU) and infant (800 IU) vitamin D supplementation attained a serum 25(OH)D > 30 ng/mL in 80% of study infants at 4 months of age.

Vitamin D status at birth was associated with sun exposure according to seasonal variation during pregnancy. In addition, circulating 25(OH)D levels are influenced by ethnicity, genetic factors and maternal factors such as prenatal vitamin D intake and gestational diabetes mellitus [27, 28]. Many studies have revealed a substantial proportion of vitamin D deficiency in the Middle East, Hispanic women, African Americans and Asian countries during pregnancy, compared to western countries [7, 23, 29, 30]. A recent study revealing a high prevalence of severe vitamin D deficiency in the Middle East prompted us to investigate the optimal vitamin D requirement of preterm infants in different geographical locations [7]. Choi et al. [31] identified that the overall prevalence of vitamin D deficiency (<20 ng/mL) was 77.3% and the prevalence of vitamin D deficiency (<10 ng/mL) was 28.6% among pregnant women in Korea. The potential risk of vitamin D deficiency on immune status of preterm infants at birth has attracted much interest recently [32]. In addition, Park et al. [33] suggested that 91.7% of preterm neonates in Korea had 25(OH)D concentrate ion < 20 ng/mL and half of them had < 10 ng/mL at birth. Similar to previous reports [34, 35], in the present study, women with GDM had a significantly increased risk of deficient vitamin D status with cord 25(OH)D concentrations ≤ 10 ng/mL. These results suggest vitamin D deficiency is related to impaired glucose tolerance and insulin resistance. Aghajafari et al. [36] recently confirmed that women who had vitamin D insufficiency during pregnancy were at risk of developing gestational diabetes and delivering small for gestational age infants in a systematic review. Vitamin D may also interact with drugs such as antibiotics, and glucocorticoids that are used to treat sepsis and BPD, respectively in preterm infants [37]. Preterm infants with these diseases may more likely to have low vitamin D levels involved in impaired vitamin D absorption or metabolism during hospitalization. Interestingly, preterm morbidities influencing vitamin D status had no significant differences between the groups in our patients.

During vitamin D supplementation, preterm infants require close monitoring of serum calcium, phosphorus, urinary calcium excretion, urinary P excretion and renal calcification, frequently detected by ultrasonography. An intake of 800 IU per day may result in excessive serum 25(OH)D status, subsequently elevating serum calcium and increasing urinary calcium excretion. In particular, preterm infants with delayed renal maturation and disturbed mineralization are predisposed to developing hypercalciuria and nephrocalcinosis, induced by excretory immaturity of the kidneys. In one infant in our study, nephrocalcinosis was associated with hypercalciuria and excessive vitamin D levels. However, nephrocalcinosis in preterm infants has a multifactorial etiology, including low gestational age and birth weight, often in combination with severe respiratory disease, and occurs as result of an imbalance between stone-promoting and stone-inhibiting factors [38].

## Conclusion

This study focused on safety and response to the early initiation of oral vitamin D supplementation followed by underlying level of vitamin D at birth in VLBW infants. Considering the efficacy and safety of vitamin D supplementation in this study, vitamin D intake of 800 IU per day may optimize vitamin D levels during early hospitalization in VLBW infants. Relatively small number of cases and absence of information on maternal vitamin D and dietary habits for analysis are potential limitations of present study. Longitudinal extension of this study and randomized controlled studies are required to explore the efficacy and clinical benefits of daily supplementation of vitamin D in dose of 800 IU in a larger cohort.

## References

[1] Jobe AH (2016). Vitamin D for extremely preterm infants. J Pediatr.

[2] Fort P, Salas AA, Nicola T, Craig CM, Carlo WA, Ambalavanan N (2016). A comparison of 3 vitamin D dosing regimens in extremely preterm infants: a randomized controlled trial. J Pediatr.

[3] Agostoni C, Buono Core G, Carnielli VP, De Curtis M, Darmaun D, Decsi T, Domellöf M, Embleton ND, Fusch C, Genzel-Boroviczeny O (2010). Enteral nutrient supply for preterm infants: commentary from the European Society of Paediatric Gastroenterology, Hepatology and Nutrition Committee on Nutrition. J Pediatr Gastroenterol Nutr.

[4] Agarwal N, Faridi MM, Aggarwal A, Singh O (2010). Vitamin D Status of term exclusively breastfed infants and their mothers from India. Acta Paediatr.

[5] Backstrom MC, Maki R, Kuusela AL, Sievanen H, Koivisto AM, Ikonen RS, Kouri T, Maki M (1999). Randomised controlled trial of vitamin D supplementation on bone density and biochemical indices in preterm infants. Arch Dis Child Fetal Neonatal Ed.

[6] Delvin EE, Salle BL, Claris O, Putet G, Hascoet JM, Desnoulez L, Messai S, Levy E (2005). Oral vitamin A, E and D supplementation of pre-term newborns either breast-fed or formula-fed: a 3-month longitudinal study. J Pediatr Gastroenterol Nutr.

[7] Dawodu A, Nath R (2011). High prevalence of moderately severe vitamin D deficiency in preterm infants. Pediatr Int.

[8] Ross AC, Taylor CL, Yaktine AL, Del Valle HB. Dietary Reference Intakes for Calcium and Vitamin D. Dietary Reference Intakes for Calcium and Vitamin D. edn. Washington (DC): National Academies Press (US); 2011. 21796828

[9] Grant CC, Stewart AW, Scragg R, Milne T, Rowden J, Ekeroma A, Wall C, Mitchell EA, Crengle S, Trenholme A (2014). Vitamin D during pregnancy and infancy and infant serum 25-hydroxyvitamin D concentration. Pediatrics.

[10] Jobe AH, Bancalari E (2001). Bronchopulmonary dysplasia. Am J Respir Crit Care Med.

[11] Volpe JJ (2001). Perinatal brain injury: from pathogenesis to neuroprotection. Ment Retard Dev Disabil Res Rev.

[12] Bell MJ, Ternberg JL, Feigin RD, Keating JP, Marshall R, Barton L, Brotherton T (1978). Neonatal necrotizing enterocolitis. Therapeutic decisions based upon clinical staging. Ann Surg.

[13] Salafia CM, Weigl C, Silberman L (1989). The prevalence and distribution of acute placental inflammation in uncomplicated term pregnancies. Obstet Gynecol.

[14] Zipitis CS, Akobeng AK (2008). Vitamin D supplementation in early childhood and risk of type 1 diabetes: a systematic review and meta-analysis. Arch Dis Child.

[15] Camargo CA, Rifas-Shiman SL, Litonjua AA, Rich-Edwards JW, Weiss ST, Gold DR, Kleinman K, Gillman MW (2007). Maternal intake of vitamin D during pregnancy and risk of recurrent wheeze in children at 3 y of age. Am J Clin Nutr.

[16] Marjamaki L, Niinisto S, Kenward MG, Uusitalo L, Uusitalo U, Ovaskainen ML, Kronberg-Kippila C, Simell O, Veijola R, Ilonen J (2010). Maternal intake of vitamin D during pregnancy and risk of advanced beta cell autoimmunity and type 1 diabetes in offspring. Diabetologia.

[17] Nguyen TM, Guillozo H, Marin L, Tordet C, Koite S, Garabedian M (1996). Evidence for a vitamin D paracrine system regulating maturation of developing rat lung epithelium. Am J Physiol.

[18] Morley R, Carlin JB, Pasco JA, Wark JD (2006). Maternal 25-hydroxyvitamin D and parathyroid hormone concentrations and offspring birth size. J Clin Endocrinol Metab.

[19] Belderbos ME, Houben ML, Wilbrink B, Lentjes E, Bloemen EM, Kimpen JL, Rovers M, Bont L (2011). Cord blood vitamin D deficiency is associated with respiratory syncytial virus bronchiolitis. Pediatrics.

[20] Namgung R, Tsang RC (2000). Factors affecting newborn bone mineral content: in utero effects on newborn bone mineralization. Proc Nutr Soc.

[21] Nutrition and feeding of preterm infants. Committee on Nutrition of the Preterm Infant, European Society of Paediatric Gastroenterology and Nutrition. *Acta Paediatr Scand Suppl.* 1987, 336:1–14. 3118635

[22] Abrams SA, Committee on N (2013). Calcium and vitamin d requirements of enterally fed preterm infants. Pediatrics.

[23] Natarajan CK, Sankar MJ, Agarwal R, Pratap OT, Jain V, Gupta N, Gupta AK, Deorari AK, Paul VK, Sreenivas V (2014). Trial of daily vitamin D supplementation in preterm infants. Pediatrics.

[24] Morrison NA, Qi JC, Tokita A, Kelly PJ, Crofts L, Nguyen TV, Sambrook PN, Eisman JA (1994). Prediction of bone density from vitamin D receptor alleles. Nature.

[25] Carpenter TO, Zhang JH, Parra E, Ellis BK, Simpson C, Lee WM, Balko J, Fu L, Wong BY, Cole DE (2013). Vitamin D binding protein is a key determinant of 25-hydroxyvitamin D levels in infants and toddlers. J Bone Miner Res.

[26] Navas-Nazario A, Li FY, Shabanova V, Weiss P, Cole DE, Carpenter TO, Bazzy-Asaad A (2014). Effect of vitamin D-binding protein genotype on the development of asthma in children. Ann Allergy Asthma Immunol.

[27] Levin GP, Robinson-Cohen C, de Boer IH, Houston DK, Lohman K, Liu Y, Kritchevsky SB, Cauley JA, Tanaka T, Ferrucci L (2012). Genetic variants and associations of 25-hydroxyvitamin D concentrations with major clinical outcomes. JAMA.

[28] Nassar N, Halligan GH, Roberts CL, Morris JM, Ashton AW (2011). Systematic review of first-trimester vitamin D normative levels and outcomes of pregnancy. Am J Obstet Gynecol.

[29] Dawodu A, Wagner CL (2007). Mother-child vitamin D deficiency: an international perspective. Arch Dis Child.

[30] Signorello LB, Shi J, Cai Q, Zheng W, Williams SM, Long J, Cohen SS, Li G, Hollis BW, Smith JR (2011). Common variation in vitamin D pathway genes predicts circulating 25-hydroxyvitamin D Levels among African Americans. PLoS One.

[31] Choi R, Kim S, Yoo H, Cho YY, Kim SW, Chung JH, Oh SY, Lee SY (2015). High prevalence of vitamin D deficiency in pregnant Korean women: the first trimester and the winter season as risk factors for vitamin D deficiency. Nutrients.

[32] Sava F, Treszl A, Hajdu J, Toldi G, Rigo J, Jr., Tulassay T, Vasarhelyi B. Plasma vitamin D levels at birth and immune status of preterm infants. Immunobiology. 2016;221(11):1289-1292. 10.1016/j.imbio.2016.06.001 27318428

[33] Park SH, Lee GM, Moon JE, Kim HM (2015). Severe vitamin D deficiency in preterm infants: maternal and neonatal clinical features. Korean J Pediatr.

[34] Soheilykhah S, Mojibian M, Rashidi M, Rahimi-Saghand S, Jafari F (2010). Maternal vitamin D status in gestational diabetes mellitus. Nutr Clin Pract.

[35] Mojibian M, Soheilykhah S, Fallah Zadeh MA, Jannati Moghadam M (2015). The effects of vitamin D supplementation on maternal and neonatal outcome: a randomized clinical trial. Iran J Reprod Med.

[36] Aghajafari F, Nagulesapillai T, Ronksley PE, Tough SC, O’Beirne M, Rabi DM (2013). Association between maternal serum 25-hydroxyvitamin D level and pregnancy and neonatal outcomes: systematic review and meta-analysis of observational studies. BMJ.

[37] Mazahery H, von Hurst PR (2015). Factors affecting 25-hydroxyvitamin D concentration in response to vitamin D supplementation. Nutrients.

[38] Hein G, Richter D, Manz F, Weitzel D, Kalhoff H (2004). Development of nephrocalcinosis in very low birth weight infants. Pediatr Nephrol.

